# Crack-MsCGA: A Deep Learning Network with Multi-Scale Attention for Pavement Crack Detection

**DOI:** 10.3390/s25082446

**Published:** 2025-04-12

**Authors:** Guoxi Liu, Xiaojing Wu, Fei Dai, Guozhi Liu, Lecheng Li, Bi Huang

**Affiliations:** 1 College of Big Data and Intelligent Engineering, Southwest Forestry University, Kunming 650224, China; guoxiliu@swfu.edu.cn (G.L.); awu@swfu.edu.cn (X.W.); daifei@swfu.edu.cn (F.D.); lecheng@swfu.edu.cn (L.L.); 2 School of Computer Science and Engineering, South China University of Technology, Guangzhou 510641, China; csliuguozhi@mail.scut.edu.cn

**Keywords:** pavement crack detection, multi-scale attention fusion, multi-scale crack detection, small-scale crack detection

## Abstract

Pavement crack detection is crucial for ensuring road safety and reducing maintenance costs. Existing methods typically use convolutional neural networks (CNNs) to extract multi-level features from pavement images and employ attention mechanisms to enhance global features. However, the fusion of low-level features introduces substantial interference, leading to low detection accuracy for small-scale cracks with subtle local structures and varying global morphologies. In this paper, we propose a computationally efficient deep learning network with CNNs and multi-scale attention for multi-scale crack detection, named Crack-MsCGA. In this network, we avoid fusing low-level features to reduce noise interference. Then, we propose a multi-scale attention mechanism (MsCGA) to learn local detail features and global features from high-level features, compensating for the reduced detailed information. Specifically, first, MsCGA employs local window attention to learn short-range dependencies, aggregating local features within each window. Second, it applies a cascaded group attention mechanism to learn long-range dependencies, extracting global features across the entire image. Finally, it uses a multi-scale attention fusion strategy based on Mixed Local Channel Attention (MLCA) selectively to fuse local features and global features of pavement cracks. Compared with five existing methods, it improves the AP@50 by 11.3% for small-scale, 8.1% for medium-scale, and 5.9% for large-scale detection over the state-of-the-art methods in the DH807 dataset.

## 1. Introduction

Cracks are widely considered an important indicator of road surface degradation. Detection of pavement cracks is a key step in assessing pavement health, conducting preventive maintenance, and ensuring driving safety. If the most common pavement distresses are not repaired in time, cracks can cause more serious damage to the pavement structure with the infiltration of water. Recently, many studies have applied deep learning to detect pavement cracks and achieved great success due to their exceptional ability to automatically extract pavement crack features from large-scale data [1,2,3,4].

In these deep learning-based models, pure CNN models (i.e., YOLOv5l [2] and Faster-RCNN-R50 [5]) and their improved versions are firstly employed to detect pavement cracks. Xing et al. [3] improved the YOLOV5 deep learning model by incorporating a bidirectional feature pyramid network (BIFPN) to fuse multi-level features for pavement crack detection. Liang el al. [4] proposed an automatic approach using multi-source image fusion (MSFSD) based on visible light and infrared sensors, with a generative adversarial network for urban pavement crack detection.

Subsequently, many attention mechanisms are added to CNN models for further capturing long-range dependencies of pavement cracks. Yang et al. [6] proposed an attention fusion block to integrate multi-scale features and suppress interference from complex backgrounds, thereby enhancing the accuracy of pavement crack detection. Yao et al. [7] proposed a pavement crack detection method based on YOLOv5, utilizing channel squeeze and excitation (SCSE) [8] and a convolutional block attention module (CBAM) [9] to optimize model performance, and investigated the impact of “how” and “where” to add attention modules, significantly enhancing detection accuracy, speed, and robustness. These methods further improve the accuracy of pavement crack detection by leveraging global attention mechanisms to learn the variable global morphology of cracks.

**Figure 1:**
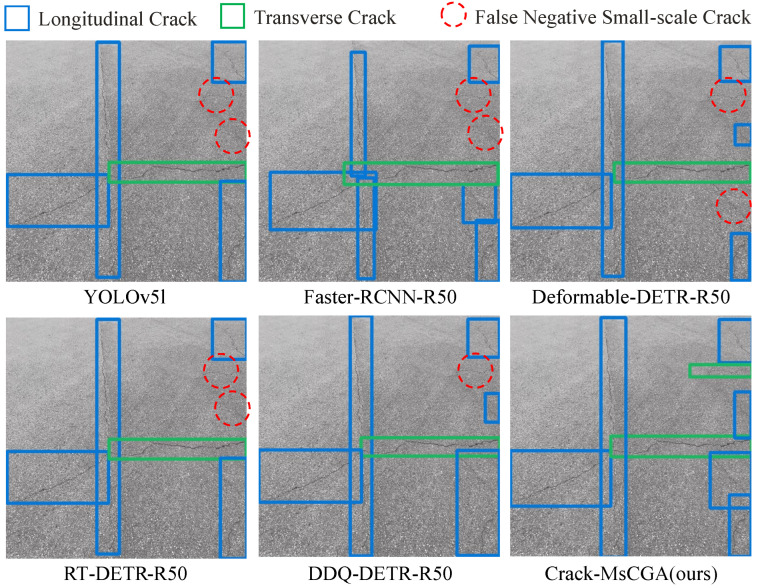
Comparison of the detection results of YOLOv5l, Faster-RCNN-R50, Deformable-DETR-R50, RT-DETR-R50, DDQ-DETR-R50, and our proposed Crack-MsCGA.

**Figure 2:**
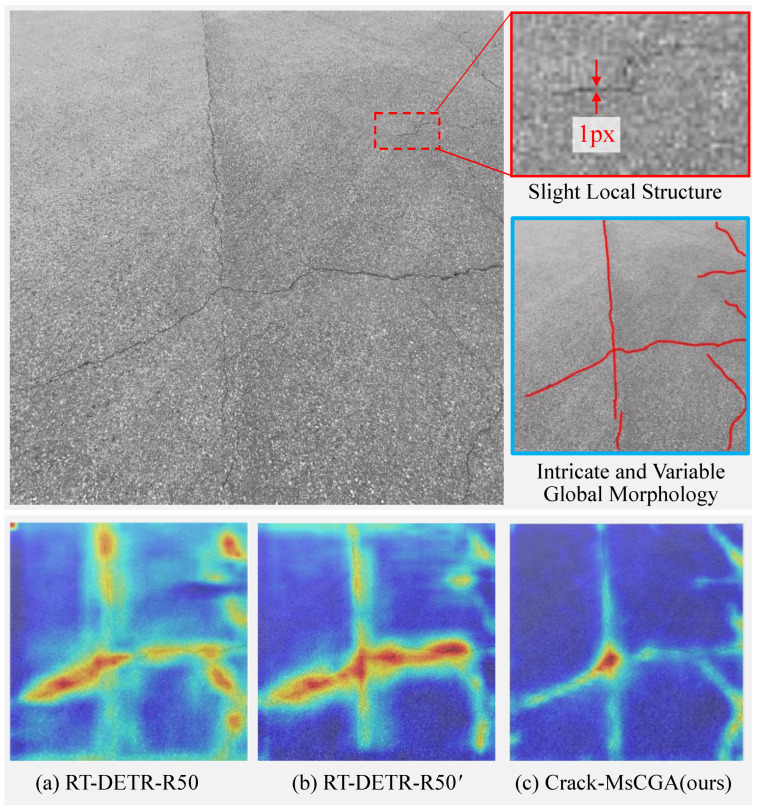
Comparison of gradient-based class activation maps (Grad-CAMs) of YOLOv5l (pure CNN models), RT-DETR-R50 (networks combining CNN and global attention mechanisms), and our proposed Crack-MsCGA.

However, as shown in Figure 1, both pure CNN models and networks that combine CNN with global attention mechanisms perform poorly in small-scale pavement crack detection. This is because those cracks exhibit different characteristics at different views: from the global viewpoint, small-scale pavement cracks exhibit intricate and variable morphology in an image; from the local viewpoint, pavement cracks exhibit little structure, as shown in the top image of Figure 2. Furthermore, in the bottom image of Figure 2, we analyze the feature extraction performance using gradient-based class activation maps (Grad-CAMs) [10]. The results indicate that RT-DETR-R50 not only focuses on crack regions but also on background textures. This additional attention to background textures makes them prone to confusing background textures with the local structure of slight pavement cracks. Therefore, the key challenge in small-scale pavement crack detection is how to effectively extract the features of local structures while reducing background noise interference.

The backbone network extracts multi-level features by sequentially applying CNN layers that filter background noise from the output of the previous layer to extract crack features. As a result, higher-level features have larger receptive fields and richer semantic information, while lower-level features preserve more detailed information but also incorporate additional background noise [11]. When the local structures of pavement cracks are insignificant, incorporating lower-level features within the Path Aggregation Network (PAN) [12] not only significantly increases the computational complexity but also introduces additional noise, thereby interfering with asphalt pavement crack detection, especially for small-scale cracks. Therefore, we designed a variant of RT-DETR-R50, referred to as RT-DETR-R50’, which omits the fusion of the lowest-level features in the FPN. As shown in Figure 2b, RT-DETR-R50’ focuses less on background textures compared to RT-DETR-R50, indicating that excluding the fusion of low-level features in the FPN can reduce background noise interference in pavement crack detection. However, this modification also results in the reduced extraction of local detail features.

In this work, we propose a novel automated pavement crack detection network named Crack-MsCGA, which excludes the fusion of low-level features in the FPN to reduce background noise interference. Then, we design a Cascaded Group Attention Fusion (MsCGA) block to capture the local structures of slight pavement cracks, thereby enhancing local detail features. MsCGA block can extract and enhance both local features and global features of pavement cracks at different scales. Specifically, local window attention is first employed to aggregate local features, capturing the local structure by learning short-range dependencies (i.e., the interrelationships between elements within the window). Subsequently, a global cascaded group attention mechanism is applied to further capture global features, capturing variable global morphology by learning long-range dependencies (i.e., the interrelationships between elements across the entire image). Finally, we propose a multi-scale attention fusion strategy based on Mixed Local Channel Attention (MLCA) to selectively integrate both local and global features.

The main contributions of this paper can be summarized as follows:

- We propose Crack-MsCGA, which is a computationally efficient pavement crack detection network. Crack-MsCGA can detect large-scale cracks, medium-scale cracks, and small-scale cracks with insignificant structure and variable morphology.
- We design an MsCGA block, which can effectively extract both local and global features of pavement cracks by learning short-range and long-range dependencies. Moreover, the block selectively fuses local features and global features through a multi-scale attention fusion strategy based on Mixed Local Channel Attention (MLCA).
- We conduct extensive experiments on the various settings and compare Crack-MsCGA with five existing methods to show its effectiveness. The results show that Crack-MsCGA significantly outperforms these methods. In addition, Crack-MsCGA is a computationally efficient method for pavement crack detection, achieving an average reduction of 32.0% in GFLOPs and 11.2% in parameters over the state-of-the-art method.

## 2. Related Work

Current mainstream methods can be classified into three categories: image processing-based detection, machine learning-based detection, and deep learning-based detection.

### 2.1. Image Processing-Based Detection and Machine Learning-Based Detection

Initially, researchers explored automated pavement crack detection through image processing-based methods. Methods such as thresholding [13], edge detection [14,15], and morphological operations [16,17] were utilized to segment and enhance crack regions, while local binary patterns (LBPs) [18] and Gabor filters [19] were employed for texture- and frequency-based feature extraction. Additionally, shape-based approaches [20] and tree-structured algorithms [21] aim to capture geometric and hierarchical characteristics of cracks. These methods often face challenges with varying lighting conditions, shadows, and complex backgrounds, which can result in poor robustness in crack detection.

More recently, machine learning-based algorithms, such as support vector machines (SVMs) [22] and random forests [23], have been employed to classify and detect cracks more accurately. These algorithms can learn from labeled datasets, improving their ability to distinguish between cracks and other pavement features. However, they require extensive feature engineering and are constrained by the quality and quantity of the training data.

### 2.2. Deep Learning-Based Detection

The advancement of neural networks is propelling computer vision forward remarkably, significantly improving the accuracy of tasks like image classification, object detection, and semantic segmentation. These developments make the automation of pavement crack detection possible.

Leveraging the powerful feature extraction capabilities of CNN networks, many pure CNN-based models have been employed for pavement crack detection. Li et al. [24] proposed a fusion model combining grid-based classification and box-based detection to enhance the accuracy and efficiency of asphalt pavement crack identification. Mayya et al. [25] proposed a triple-stage framework combining YOLO-ensemble, MobileNetV2U-net, and spectral clustering to effectively detect cracks in stone masonry, addressing the challenges of diverse crack scales and morphologies. Raushan et al. [26] leveraged the YOLO network to effectively detect damage in concrete structures, addressing challenges posed by multi-feature backgrounds. Deng et al. [27] proposed a crack-boundary refinement framework to achieve precise segmentation of high-resolution crack images, enhancing both the safety and efficiency of UAV-based bridge inspection processes. Chu et al. [28] proposed a multi-scale crack feature extraction network (MsCFEN) with the embedment of the strip pooling operation to achieve a balance between segmentation accuracy and GPU memory consumption, this being a challenge when segmenting crack images with resolutions exceeding 4K. Compared to traditional image processing-based methods and machine learning-based methods, deep learning approaches, particularly Convolutional Neural Networks (CNNs), achieve higher detection accuracy and robustness. This is due to the exceptional ability of CNN-based deep learning methods to automatically extract pavement crack features within large-scale data.

Many attention mechanisms have been added to CNN models to further capture long-range dependencies of pavement cracks in order to improve detection accuracy; to achieve high precision and efficiency, convolutional block attention modules and architectural optimizations have been implemented. Zhang et al. [29] transformed pavement images from one perspective to Bird’s Eye View images using binocular parallax information, employ a Unet with attention mechanisms to selectively fuse deep and shallow features for crack identification; the results demonstrated high detection accuracy for complex pavement conditions. Zhu et al. [30] proposed a novel encoder–decoder architecture that integrated a new type of hybrid attention block with residual blocks (RBs), resulting in an extremely lightweight model that achieved more accurate detection of pavement crack pixels. Dong et al. [31] achieved high precision and efficiency by integrating a convolutional block attention module and architectural optimizations. Zheng et al. [32] proposed an application-oriented multi-stage crack recognition framework that sequentially employs classification model training, semi-supervised training, pixel-level crack segmentation, and crack width measurement for comprehensive crack detection. Yang et al. [6] proposed an attention fusion block to integrate multi-scale features and suppress interference from complex backgrounds, thereby enhancing the accuracy of pavement crack detection. These models achieve significant success by incorporating attention mechanisms because these mechanisms capture the global features of pavement cracks by learning the long-range dependencies. However, their enhancement of local features is limited, and they may even diminish local features learned by CNNs [33,34,35].

Unlike these approaches, Crack-MsCGA reduces noise interference by reducing the fusion of low-level features. It then incorporates a multi-scale attention mechanism to learn both local and global features from high-level features, thereby improving the accuracy of pavement crack detection.

## 3. Methodology

In this section, we introduce first the architecture of Crack-MsCGA, and then the design of the MsCGA block.

### 3.1. Network Architecture

**Figure 3:**
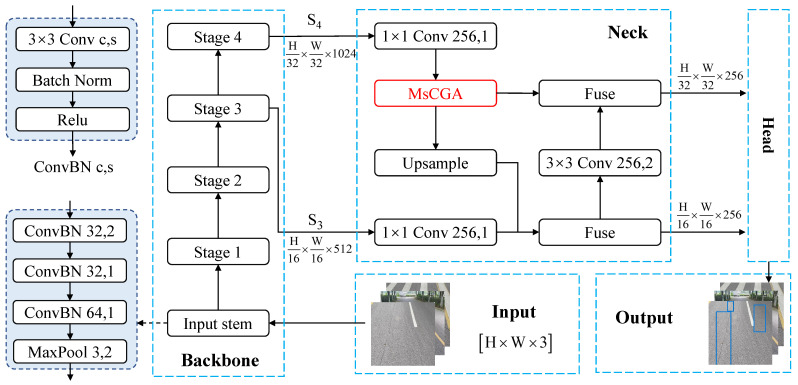
The network structure of Crack-MsCGA, where c and s in ConvBN denote the channel number and stride of the convolution, respectively. Fuse denotes the use of three consecutive fusers, whose structure is illustrated in Figure 4, to merge input features.

Crack-MsCGA is proposed to detect large-scale cracks, medium-scale cracks, and small-scale cracks with insignificant structure and variable morphology; it consists of three parts: the backbone network, the neck, and the detection head. Figure 3 shows the overall architecture of Crack-MsCGA. First, the input layer resizes the input pavement images to a size of H×W×3 and then feeds them into the backbone network, where *H* and *W* represent the height and width of the resized image, respectively. Second, the backbone network, which is an improved version of ResNet-50 [36], is used to extract multi-level features (i.e., high-level features and low-level features) of the resized images. Subsequently, in the neck, MsCGA is proposed to enhance high-level features and then fuse them with low-level features through a top–down and bottom–up pathway. Finally, these fused feature maps are fed into the detection head to generate the bounding boxes and classes of pavement cracks.

**Figure 4:**
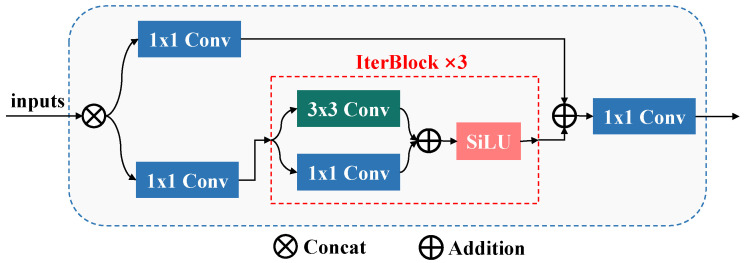
Network structure of fuser.

#### 3.1.1. The Backbone

The backbone network consists of an input stem and four subsequent stages, which can extract multi-level features of pavement images, as shown in Figure 3. Inspired by He et al. [37], three conservative ConvBN blocks are designed to replace the 7×7 convolutional layer in the input stem. The ConvBN block consists of a 3×3 convolutional layer, a batch normalization (BN) [38] layer, and a ReLU activation layer. This combination enhances the feature extracting stability of the backbone network because the batch normalization layer breaks the independence of each sample’s loss, and the ReLU activation layer improves the backbone network’s non-linear representation capabilities. Then, the ConvBN block is used to replace each 3 × 3 convolution in each stage. The output of Stage 3 is a low-level feature map, denoted as $S_{3}\in R^{\frac{H}{16}\times \frac{W}{16}\times 512}$. The output of Stage 4 is a high-level feature map, denoted as $S_{4}\in R^{\frac{H}{32}\times \frac{W}{32}\times 1024}$. Finally, $S_{3}$ and $S_{4}$ are fed into the neck.

#### 3.1.2. The Neck

The backbone network employs ResNet50 to extract multi-level features by sequentially applying CNN layers that filter background noise from the output of the previous layer to extract crack features. As a result, high-level features have larger receptive fields and richer semantic information, while low-level features preserve more detailed information but also incorporate additional background noise. Due to the insignificant local structures of slight asphalt pavement cracks, incorporating lower-level features within the Path Aggregation Network (PAN) [12] not only significantly increases the computational load but also introduces additional noise, thereby interfering with asphalt pavement crack detection, especially for small-scale cracks.

Therefore, as shown in Figure 3, we designed a unique neck structure in Crack-MsCGA. On one hand, we designed the MsCGA block to enhance and aggregate local features and to learn the global feature representations of high-level features for the purpose of enriching the detailed features and capturing the variable global morphology.

The details of the MsCGA block are described in Section 3. On the other hand, unlike conventional object detection models that integrate lower-level features (e.g., the output from stage 2), our approach only fuses the high-level feature $S_{4}$ and the low-level feature $S_{3}$ through a top–down and bottom–up pathway [12].

#### 3.1.3. The Detection Head

In Crack-MsCGA, we employ an RT-DETR [39] detection head to generate the bounding boxes and categories of pavement cracks. The RT-DETR detection head is an improved version of DETR [40]. It inherits DETR’s end-to-end detection characteristics while achieving a faster inference speed. This allows Crack-MsCGA to quickly detect the bounding boxes and categories of cracks while maintaining well-organized prediction boxes.

#### 3.1.4. Loss Function

In our Crack-MsCGA model, we use Generalized Intersection over Union Loss ($L_{GIoU}$) [41] and L1 Loss ($L_{L1}$) [42] to compute the bounding box loss, and then use Cross-Entropy Loss ($L_{CE}$) [43] to compute the classification loss. The overall loss function is formulated as follows:

(1) $$L=\alpha \cdot L_{GIoU}+\beta \cdot L_{L1}+\gamma \cdot L_{CE}$$

where α, β, and γ are hyperparameters that balance the contributions of each loss component.

### 3.2. Multi-Scale Cascaded Group Attention Fusion Block

**Figure 5:**
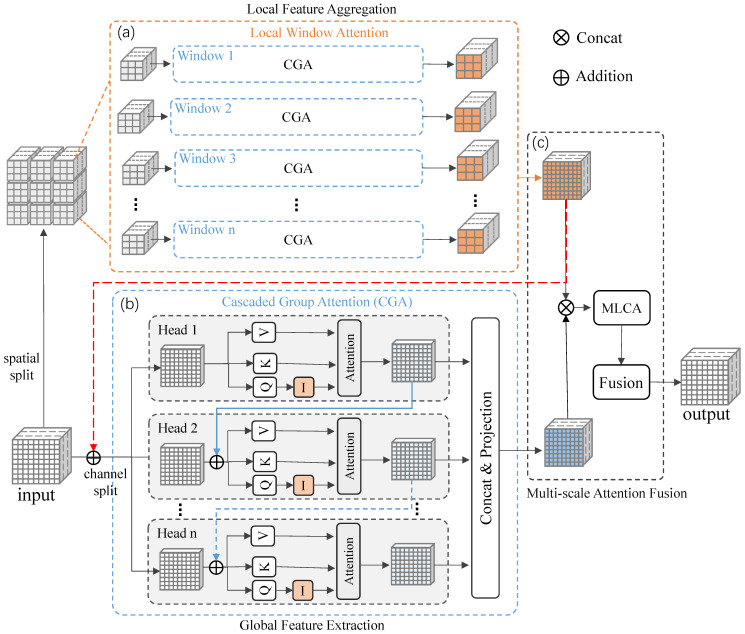
Architecture of MsCGA. It consists of three parts: the bottom branch (**b**) represents Cascaded Group Attention for global feature extraction, the top branch (**a**) represents local window attention for local feature extraction, and (**c**) indicates multi-scale attention fusion, where Attention represents scaled dot-product attention.

In this section, we elaborate the details of the Multi-scale Cascaded Group Attention Fusion (MsCGA) block, which can effectively extract and integrate both local and global features of pavement cracks. Specifically, the computation process of this block consists of three stages. During the stage of local feature aggregation, this block firstly divides the input features into multiple windows and then uses the local window attention to compute short-range dependencies within each patch, as shown in the top branch of Figure 5. During the stage of global feature extraction, this block uses the cascaded group attention to learn long-range dependencies across the entire image, as shown in the bottom branch of Figure 5. Finally, a multi-scale attention fusion strategy based on Mixed Local Channel Attention (MLCA) is proposed to selectively fuse features learned by multi-scale attention, as illustrated in Figure 5c.

#### 3.2.1. Local Feature Aggregation

We employ a local window attention mechanism to compute short-range dependencies, aggregating the local features of pavement cracks within each window patch, as shown in the top branch of Figure 5. Firstly, we spatially divide the input features map $S_{4}$ into windows, where each window has a size of ws, i.e., $S_{4}=\left\{W_{11},W_{12},W_{13},\ldots ,W_{n,n-2},W_{n,n-1},W_{n,n}\right\}$, where $n=\lceil \frac{W}{ws}\rceil$. Then, we aggregate local features of pavement cracks by using Cascaded Group Attention (CGA) [44] to compute the dependencies of each element in each patch.

(2) $${W_{ij}}^{\prime }=CGA\left(W_{ij}\right)$$

where $W_{ij}$ denotes the window patch at the i-th row and j-th column, and CGA(·) denotes using Cascaded Group Attention to compute dependencies of the input patch. Finally, we combine the aggregated features within windows to obtain local features $F_{L}\in R^{\frac{H}{32}\times \frac{W}{32}\times 256}$.

Traditional multi-head self-attention (MHSA) mechanisms suffer from high computational costs due to each head computing attention across all channels. To improve the computational efficiency of MsCGA, we choose CGA to calculate self-attention for reducing redundant computations. In fact, CGA is an advanced MHSA, which divides the input features map into multiple channel groups and assigns these channel groups to different attention heads. This attention mechanism enables each attention head to process a unique channel group of the input feature map, thereby enhancing the diversity of the attention maps. Formally, the computation process of CGA can be formulated as follows:

Let *h* be the total number of attention headers. The input feature map *F* with *C* channels is divided into *h* channel groups, with each group containing $\frac{C}{h}$ channels, i.e., $F\;=\;\left[F_{1},F_{2},\ldots ,F_{h}\right]$. Here, $F_{k}$ denotes the k-th split of the input feature, where 1≤k≤h. Then, we utilize *h* attention heads to individually calculate the self-attention for each channel group. Finally, we concatenate the output features of each self-attention head and employ a linear layer to project its dimensions to align with the input feature map.

(3) $${\bar{F}}_{k}=Attention\left(F_{k}W_{k}^{Q},F_{k}W_{k}^{K},F_{k}W_{k}^{V}\right)$$

(4) $$CGA(F)=Concat{\left[{\bar{F}}_{k}\right]}_{k=1:h}W^{P}$$

where $W_{k}^{Q}$, $W_{k}^{K}$, and $W_{k}^{V}$ are learnable parameters that project each group of feature map to different subspaces, Attention(·) denotes the calculation of self-attention [45], and $W^{P}$ denotes a linear layer that projects the concatenated output feature map dimensions to align with the input feature map.

As illustrated in Figure 5b, we cascade the output features of each preceding attention head into the input features of the subsequent attention head, thereby enhancing the input information for attention computation. Specifically, the computation method for the input of each attention head is as follows:

(5) $$F_{k}^{\prime }=F_{k}+{\bar{F}}_{k-1},1<k⩽h$$

where $F_{k}^{\prime }$ denotes the input feature of the *kk*-th head and ${\bar{F}}_{k-1}$ denotes the output feature of the (k−1)-th attention head.

#### 3.2.2. Global Feature Extraction

We employ CGA to capture intricate and variable global features by learning long-range contextual information across the entire image, based on the aggregated local features of cracks in the previous process. Specifically, as shown in the bottom branch of Figure 5, we first add the local feature map aggregated by the local window attention mechanism with $S_{4}$, enriching the input information of the global attention:

(6) $$\bar{S_{4}}=S_{4}+F_{L}$$

Then, we use cascaded group attention to capture global features $F_{G}\in R^{\frac{H}{32}\times \frac{W}{32}\times 256}$ from those features by learning long-range contextual information across the entire image:

(7) $$F_{G}=CGA\left(\bar{S_{4}}\right)$$

#### 3.2.3. Multi-Scale Attention Fusion

In the previous section, we utilized multi-scale attention to capture both short-range and long-range dependencies for extracting local and global features. In this section, we propose a multi-scale attention fusion strategy to selectively integrate these features, as shown in Figure 5c. Specifically, we first concatenate the local feature map and global feature map:

(8) $$F_{M}=Concat\left(F_{L},F_{G}\right)$$

where Concat(·) refers concatenating input multi-scale attention features. Then, we use Mixed Local Channel Attention (MLCA) [46] to reweight different channels and various regions within the same channel of the concatenated features:

(9) $$F_{M}=MLCA\left(F_{M}\right)$$

where MLCA(·) denotes using MLCA to reweight features. Finally, we employ a CNN-based fuser to integrate them.

(10) $$F_{M}=Fuse\left(F_{M}\right)$$

where Fuse(·) denotes employing a fuser to integrate features. Figure 4 shows the network structure of the fuser.

## 4. Experiments

In this section, we first introduce the experimental settings in Section 4.1. Secondly, we conduct a comparative analysis of our proposed Crack-MsCGA and five other advanced crack detection models from the perspectives of multi-scale crack detection, efficiency, and visualization in Section 4.2. Thirdly, we conduct a comparative analysis of our proposed MsCGA block with a control group without the attention mechanism and with two classical attention mechanisms and three state-of-the-art attention mechanisms in Section 4.3. Finally, we design some comparative to validate the rationality of the Crack MSCGA design in Section 4.6.

### 4.1. Experimental Settings

#### 4.1.1. Experimental Environment

The experimental environment was as follows: Windows11, Python 3.11.6, Pytorch 2.1.0, CUDA11.8, Intel(R) Xeon(R) Silver 4314, NVIDIA RTX A6000.

#### 4.1.2. Datasets

**Figure 6:**
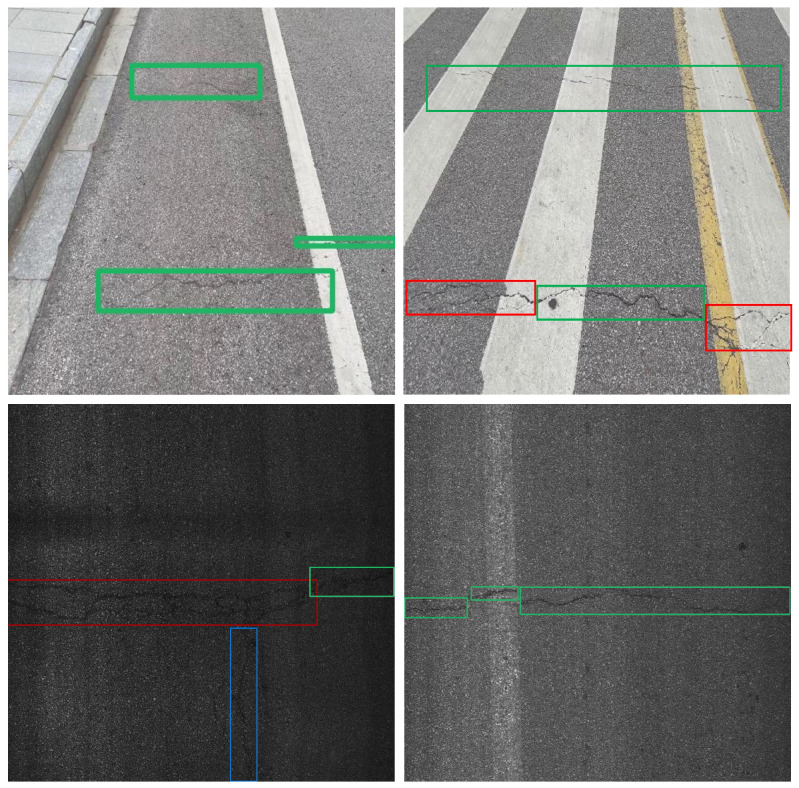
Some images from the datasets and their corresponding labels: green bounding boxes indicate a Transverse Crack (TC), blue bounding boxes indicate a Longitudinal Crack (LC), and red bounding boxes indicate a Block Crack (BC).

We conducted experiments on a public dataset (RDD2022-China-MotorBike, China-M) and a private dataset (DH807) to validate the effectiveness of our proposed method. The China-M dataset was captured from a rear-to-front perspective, as shown in the first row of Figure 6, while DH807 was captured from a top--down perspective, as shown in the second row of Figure 6.

The public China-M [47] dataset is a more common dataset for pavement distress detection, including four types of pavement damage: Longitudinal Cracks, Transverse Cracks, Block Cracks, and Potholes. We constructed a new dataset based on RDD2022-China-MotorBike to test the effectiveness of our proposed method in the task of crack detection. Specifically, we first deleted images without cracks from the dataset. Then, we removed non-crack labels and manually corrected incorrect labels. Finally, we randomly divided the dataset into a training set and a test set in a 7:3 ratio, yielding 1290 training images and 560 test images. Each image is a grayscale image with a resolution of 512×512 pixels. The first row of Figure 6 shows two images from the dataset.

The DH807 dataset is a new dataset proposed by us. Specifically, we used a pavement image collection vehicle developed by Wuhan Optics Valley Zoyon Science and Technology to collect pavement images from a top–down perspective in Dehong Prefecture, Yunnan Province, China. This collection vehicle was equipped with advanced high-speed cameras, shooting control algorithms, and postprocessing alignment algorithms, ensuring continuous high-definition pavement images at speeds ranging from 0 to 100 km/h. Subsequently, we selected 807 images containing cracks from the collected pictures and annotated them using LabelImg. These images were then divided into a training set and a test set in a 7:3 ratio. Second row of Figure 6 shows two images from the dataset.

#### 4.1.3. Evaluation Metrics

In object detection, several metrics are used to evaluate the accuracy of a model. True Positives (*TP*) are the correctly detected objects, i.e., the objects that are correctly identified by the model. False Positives (*FP*) are the incorrectly detected objects, i.e., the objects that the model incorrectly identifies as present. False Negatives (*FN*) are the objects that the model fails to detect, i.e., the objects that are present but not identified by the model. Precision (*P*) is the ratio of *TP* to the sum of *TP* and *FP*:

$$P=\frac{TP}{TP+FP}$$

Recall (*R*) is the ratio of *TP* to the sum of *TP* and *FN*:

$$R=\frac{TP}{TP+FN}$$

*F*1 score is the harmonic mean of precision and recall, primarily used to evaluate the balance between precision and recall in classification or detection tasks. Its formula is as follows:

$$F1=\frac{2\times P\times R}{P+R}$$

To comprehensively analyze the balance of crack detection across different scales, this study utilizes the mean *F*1 score (*mF*1), which is calculated as the average of the *F*1 scores for small, medium, and large scales, to evaluate the model’s balance between precision and recall:

$$mF1=\frac{1}{S}\sum_{i=1}^{S}F1_{i}$$

where (*S*) is the number of scales and $F1_{i}$ is the *F*1 score for the (*i*)-th scale. Average Precision (*AP*) is the area under the precision–recall curve, summarizing the precision and recall at different threshold levels. hlAP@50 is a specific instance of *AP* where the Intersection over Union (IoU) threshold is set to 0.50. This means that a predicted bounding box is considered a True Positive if its IoU with the ground truth box is greater than or equal to 0.50. **Mean Average Precision (*mAP*)** is a commonly used metric in object detection that averages the *AP* across all classes. It provides a single performance measure that accounts for both precision and recall across different object categories. The *mAP* is calculated as follows:

$$mAP=\frac{1}{N}\sum_{i=1}^{N}AP_{i}$$

where (*N*) is the number of classes and $AP_{i}$ is the Average Precision for the (*i*)-th class.

In addition to accuracy metrics, some metrics are used to evaluate the inference speed and computational complexity of the models. In this study, Frames Per Second (FPS) is utilized to assess the inference speed, while GFLOPs and parameters are employed to evaluate the computational complexity of the models.

#### 4.1.4. Baselines

In our study, we conduct a comparative analysis of our method Crack-DETR with five existing models: YOLOv5l [2], YOLOv5l-ST-BIFPN [3], UM-YOLOl [48], Faster-RCNN-R50 [5], Deformable-DETR-R50 [49], DDQ-DETR-R50 [50], and RT-DETR-R50 [39]. Among them, YOLOv5l and Faster-RCNN-R50 are purely CNN-based models, while Deformable-DETR-R50, DDQ-DETR-R50, and RT-DETR-R50 are end-to-end detection models that leverage attention mechanisms. Additionally, YOLOv5-ST-BIFPN and UM-YOLOl are improved versions of YOLOv5 and YOLOv8 tailored for pavement crack detection. To ensure a fair comparison, we implemented YOLOv5-ST-BIFPN and UM-YOLOl by following the methods outlined in the respective papers, based on the YOLOv5 and YOLOv8 variants with parameters most similar to Crack-MsCGA (i.e., YOLOv5l and YOLOv8l).

**YOLOv5l:** YOLOv5l is a classical single-stage object detection model known for its speed and efficiency. It is part of the YOLO (You Only Look Once) family, which emphasizes real-time detection with good accuracy.

**YOLOv5l-ST-BIFPN:** YOLOv5l-ST-BIFPN is an improved version of YOLOv5l, integrating the Swin Transformer block and Bidirectional Feature Pyramid Network (BIFPN) into YOLOv5. These enhancements enable it to achieve outstanding performance in UAV pavement crack detection.

**UM-YOLOl:** UM-YOLOl is an enhanced version of YOLOv8l. It integrates an Efficient Multiscale Attention (EMA) module into the backbone’s C2f module, employs a Bi-FPN fusion mechanism in the neck to improve feature aggregation, and leverages GSConv—a lightweight convolutional network—for more efficient convolution operations.

**Faster-RCNN-R50:** Faster-RCNN-R50 is a classical two-stage object detection model. It first generates region proposals and then refines these proposals for final object detection and classification.

**Deformable-DETR-R50:** Deformable DETR (DEtection TRansformer) with a ResNet-50 backbone integrates deformable convolutions into the DETR framework. This model improves upon the original DETR by enhancing its ability to handle small objects and varying object scales through deformable attention, leading to better performance in object detection tasks.

**DDQ-DETR-R50:** DDQ-DETR-R50 stands for Dense Distinct Query for end-to-end object detection with a ResNet-50 backbone. This model leverages dense distinct queries in a transformer-based framework, enhancing its ability to detect objects with varying scales and shapes.

**RT-DETR-R50:** RT-DETR-R50 denotes Real-Time DETR with a ResNet-50 backbone. It aims to bring the benefits of the DETR model, including its transformer-based architecture and attention mechanisms, to real-time applications. RT-DETR-R50 balances the high accuracy of transformer-based detection with the speed necessary for real-time inference.

#### 4.1.5. Hyperparameter Settings

For a fair comparison, we carefully tune the hyperparameters of all baselines to report their best performance. The number of epochs is set to 300, the learning rate is set to 1 $\times \;10^{-4}$, the weight decay used in the local optimizer is set to 1 $\times \;10^{-4}$, the batch size for training is set to 16, and the input image size is 640×640. In the Local Window Attention of MsCGA block, the size of each window (ws) is set to 5. For the loss function, the hyperparameters α, β, and γ are set to 2, 1, and 1, respectively.

### 4.2. Detection Performance

In this section, we evaluate Crack-MsCGA on the China-M dataset and DH807 dataset, then compare it with seven baselines.

#### 4.2.1. Performance on the China-M Dataset

The performance of the methods on China-M is summarized in Table 1, with AP@50 metrics reported for small, medium, and large objects as well as the mean of all scales.

As shown in Table 1, we first analyze the detection performance under varying scales of cracks, i.e., small, medium, and large. Specifically, for the most challenging small-scale cracks with slight structures and variable morphologies, Crack-MsCGA outperforms the baselines with an average improvement of 11.7% (i.e., improving AP@50 to 11.3%, 11.1%, 8.3%, 14.9%, 16.1%, 7.7%, and 12.2% on YOLOv5l, YOLOv5l-ST-BIFPN, UM-YOLOl, Faster-RCNN-R50, Deformable-DETR-R50, DDQ-DETR-R50, and RT-DETR-R50, respectively). In particular, compared to pure CNN models (i.e., YOLOv5l and Faster-RCNN-R50), Crack-MsCGA achieves improvements of up to 11.3% and 14.9% in detection performance, respectively. Models based on CNNs with attention mechanisms (e.g., UM-YOLOl, DDQ-DETR-R50, and RT-DETR-R50) show improved accuracy in detecting large-scale and medium-scale cracks compared to pure CNN models. However, the improvement in detecting small-scale cracks remains relatively limited. Overall, Crack-MsCGA achieved the highest accuracy across all scales. Even when compared to the closest method (i.e., DDQ-DETR-R50), Crack-MsCGA demonstrated improvements of 7.7%, 0.5%, and 1.6% in detecting small-scale, medium-scale, and large-scale cracks, respectively. In addition, the mF1 score of Crack-MsCGA is higher than that of all other models, achieving the top rank. This indicates that Crack-MsCGA has a stronger balance between precision and recall. On the one hand, these improvements are attributed to MsCGA’s ability to avoid the noise interference present in low-level features. On the other hand, they are credited to the MsCGA block, which employs local window attention to aggregate local features by learning short-range dependencies within the patch. This enhances the feature representation capability for small-scale cracks with subtle structures and variable morphologies.

Overall, the results validate the effectiveness of Crack-MsCGA in improving the detection performance of pavement cracks on the China-M dataset, making it a robust and reliable solution for automated pavement crack detection.

#### 4.2.2. Performance on DH807 Dataset

As shown in Table 2, the proposed Crack-MsCGA achieved the highest average AP@50 across three scales, reaching 60.3%, which is an improvement of 2.5% over the closest model, RT-DETR-R50, on the DH807 dataset. Specifically, for the most challenging slight small-scale cracks with insignificant structures and variable morphologies, Crack-MsCGA outperforms the baselines with an average improvement of 11.3% (i.e., improving AP@50 to 7.3%, 9.1%, 10.9%, 17.5%, 16.3%, 12.1%, and 6.0% on YOLOv5l, YOLOv5l-ST-BIFPN, UM-YOLOl, Faster-RCNN-R50, Deformable-DETR-R50, DDQ-DETR-R50, and RT-DETR-R50, respectively). For the medium-scale cracks, Crack-MsCGA achieved 6.7% average improvement on AP@50. For the large-scale cracks, Crack-MsCGA achieved a 5.9% average improvement on AP@50. From the perspective of the F1 score, Crack-MsCGA still achieved the best performance on the DH807 dataset, whereas DDQ-DETR-R50, which performs well on the China-M dataset, experienced a significant drop in mF1 score on the DH807 dataset. This indicates that Crack-MsCGA exhibits better robustness compared to the other models. These improvements further illustrate the effectiveness of MsCGA in enhancing the detection of small-scale pavement cracks. The MsCGA block uses local window attention to aggregate local features by learning short-range dependencies within the patch, enhancing the feature representation abilities for small-scale cracks with delicate structures and varying morphologies.

#### 4.2.3. Analysis on Efficiency

The computational cost and number of parameters are crucial reference metrics for deploying models on portable devices with integrated sensors because computational power and memory are highly limited on such devices. Therefore, we compare the computational cost (GFLOPs) and the number of parameters of Crack-MsCGA and other models to analyze their efficiency on portable devices with integrated sensors. The experimental results, as summarized in Table 3, clearly demonstrate the superior performance of our proposed method, Crack-MsCGA, compared to other techniques.

Firstly, in terms of computational efficiency, Crack-MsCGA requires only 44.5 GFLOPs, the lowest among the compared methods, achieving a reduction of on average 32.0%. This efficiency is crucial for real-time applications where computational resources and processing time are limited.

Secondly, Crack-MsCGA has the fewest parameters, with only 38.5 G, which not only reduces the model’s complexity but also minimizes the memory footprint. This makes it highly suitable for deployment on devices with limited memory, such as portable devices with integrated sensors.

Thirdly, to further explore the practical inference speed of Crack-MsCGA, we conducted FPS tests on different platforms and under various floating point precisions. The experimental results are shown in Table 4. Analysis of these results reveals that the inference speed of YOLOv5l leads all other models under all conditions. Our proposed Crack-MsCGA follows closely, achieving second place in all metrics. Overall, with FP16 precision on the NVIDIA RTX 3060, Crack-MsCGA provides an average FPS improvement of 44.4% compared to other models. This indicates that the inference speed of Crack-MsCGA outperforms most real-time object detection models.

Overall, Crack-MsCGA’s parameter count and inference computational cost outperform all other models, while its inference speed is second only to YOLOv5l. It can meet real-time inference requirements in most scenarios, making it a highly effective solution for practical applications.

#### 4.2.4. Gradient-Based Class Activation Maps Visualization

**Figure 7:**
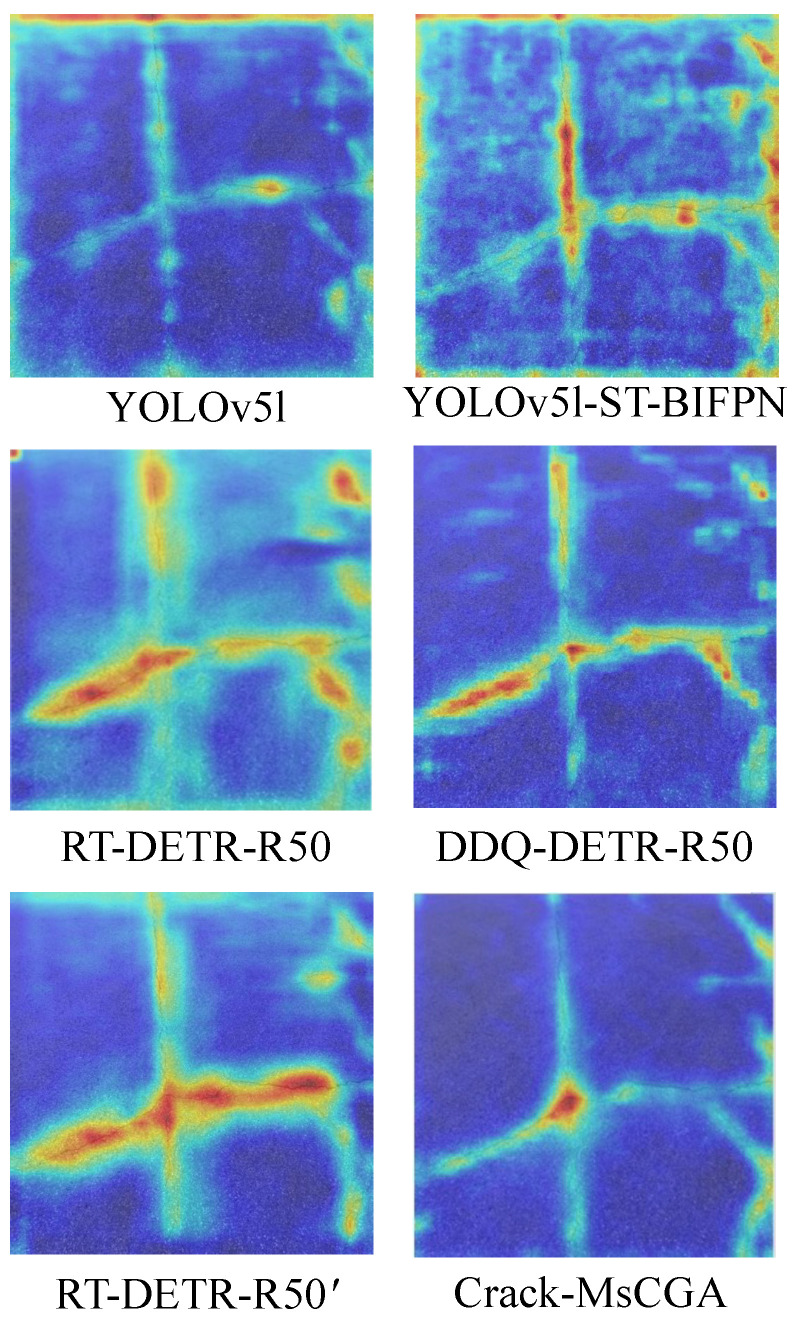
The gradient-based class activation maps of the output convolutional layers of the neck of Crack-MsCGA and other models.

In this section, we use Grad-CAM++ to generate gradient-based class activation maps to analyze the effectiveness of the backbone network and neck in extracting pavement crack features in Crack-MsCGA and other models, as shown in Figure 7.

From Figure 7, we can observe that the pure CNN models YOLOv5l and YOLOv5l-ST-BIFPN not only focus on the crack regions but also pay considerable attention to the pavement background textures. In contrast, the CNN-with-attention-based models RT-DETR-R50 and DDQ-DETR-R50, thanks to the incorporation of a global attention mechanism, significantly reduce the focus on background regions compared to the pure CNN models and place greater emphasis on the overall continuity of the cracks. Furthermore, we designed a variant of RT-DETR-R50, named RT-DETR-R50’, which removes the fusion of low-level features. Compared with RT-DETR-R50, this variant further reduces attention on background regions, but it neglects some local detail features. Crack-MsCGA builds on the removal of low-level feature fusion by using MsCGA to aggregate both the local detail features and global features of cracks at multiple scales, thereby further reducing focus on background regions while effectively concentrating on the crack areas.

#### 4.2.5. Visualization of Prediction Results

**Figure 8:**
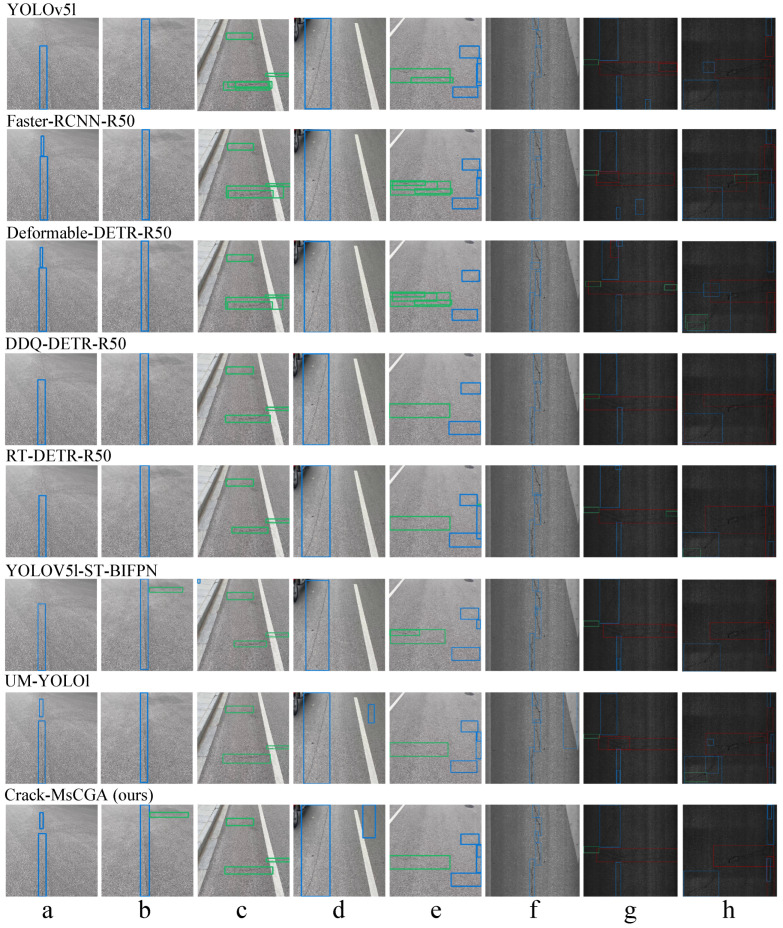
Prediction performance visualizations of Crack-MsCGA and baseline models, with samples (**a**–**e**) selected from the China-M dataset and samples (**f**–**h**) chosen from the DH807 dataset.

In Figure 8, we present the prediction results of Crack-MsCGA and other comparative models for randomly selected samples (a–h) from the test set.

From Figure 8a,b,e, it is evident that Crack-MsCGA can detect slight small-scale cracks with insignificant structure and variable morphology. This capability is attributed to MsCGA, where local window attention focuses on the interrelationships within local windows, enhancing the model’s ability to capture features of slight small-scale pavement cracks with insignificant structures and variable morphologies. Figure 8d–f illustrate that, compared to pure CNN models, models employing global attention mechanisms—such as Deformable-DETR-R50, DDQ-DETR-R50, and RT-DETR-R50—tend to overlook slight cracks and critical local details, such as endpoints. This phenomenon occurs because the features learned by the global attention mechanism weaken local features. In contrast, Crack-MsCGA effectively addresses this issue. On one hand, Crack-MsCGA enhances both local and global features by learning short-range and long-range dependencies. On the other hand, in the detection of slight small-scale cracks, the multi-scale attention fusion strategy can selectively retain more local features.

From Figure 8c,e,g, it is evident that pure CNN models such as YOLOv5l and Faster-RCNN-R50 often produce cluttered bounding boxes, even with the application of Non-Maximum Suppression (NMS) as a post-processing step. In contrast, the detection results from DDQ-DETR-R50, RT-DETR-R50, and Crack-MsCGA exhibit more orderly bounding boxes. This improvement is attributed to the use of global attention mechanisms in DDQ-DETR-R50, RT-DETR-R50, and Crack-MsCGA, which can capture long-range dependencies among input features.

By analyzing Figure 8e,h, it is observed that Crack-MsCGA tends to overly focus on local details when handling large-scale and structurally complex cracks. This results in scenarios where a single crack is detected as two smaller ones, or part of a large crack is identified as a small, separate crack. This issue is even more pronounced in pure CNN models like Faster-RCNN-R50 and YOLOv5l. However, models utilizing global attention mechanisms, such as DDQ-DETR-R50 and RT-DETR-R50, alleviate this problem to some extent. This suggests that when dealing with large-scale, complex crack structures, MsCGA may face limitations in adequately capturing global features.

From the above analysis, we can conclude that Crack-MsCGA effectively utilizes local window attention mechanisms to capture short-range dependencies of pavement cracks, aggregating fine-grained local features, which enables it to detect slight small-scale pavement cracks. Additionally, Crack-MsCGA employs cascaded group attention to capture long-range dependencies of pavement cracks for extracting intricate and variable global features, ensuring the regularity and simplicity of the prediction results. This confirms the significant role of MsCGA in improving the accuracy of pavement crack detection.

### 4.3. Comparison with Other Attention Mechanisms

In this section, we conduct comparative experiments including a control group without attention mechanisms (WOA), two classical attention mechanisms (CBAM and DAttention), and three state-of-the-art attention mechanisms (AIFI, FLA, and LSKA). For a fair comparison, we construct comparison models by replacing the MsCGA block in Crack-MsCGA with those attention mechanisms. The results are summarized in Table 5, which presents AP@50 for three scales of cracks—small, medium, and large—as well as the mean performance (mean). It is evident that our MsCGA attention outperforms all other attentions across all scales. Specifically, MsCGA achieves the highest AP@50 scores for small-scale cracks (84.8%), medium-scale cracks (86.8%), and large-scale cracks (96.3%), resulting in a mean AP@50 of 89.3%. This demonstrates the superior capability of MsCGA in accurately detecting all-scale pavement cracks.

In comparison, classical attention mechanisms, i.e., CBAM and DAttention, show relatively lower performance for small-scale crack detection than WOA, which operates without an attention mechanism. This phenomenon occurs because those attention mechanisms may diminish the local features extracted by CNNs when capturing global features. The state-of-the-art methods, AIFI, FLA, and LSKA, show improvements over WOA in medium-scale and large-scale crack detection. However, the improvements in small-scale crack detection are minimal. This is because these attention mechanisms can enhance global features by learning long-range dependencies, but their enhancement of local features is limited. It is worth noting that Crack-MsCGA achieves significant improvements across all scales. This benefits from the fact that MsCGA can not only enhance both local and global features by learning short-range and long-range dependencies, but also selectively fuse them.

### 4.4. Comparative Experiments on Where to Employ the MsCGA Block

In the neck of the Crack-MsCGA model, we only apply attention enhancement to high-level features, without enhancing low-level features. This is based on the theory that applying attention enhancement to concatenated multi-level features simultaneously is redundant because high-level features are abstractions of low-level features in generic object detection. However, there are significant differences between pavement crack detection and traditional object detection. Therefore, we set up ablation experiments to explore where employing the MsCGA block leads to the best results. As shown in Table 6, we present the computational cost and mAP@50 of Crack-MsCGA and its three variants. The WOA variant does not use the MsCGA block to enhance features. The AllLevel variant uses the MsCGA block to enhance both high-level and low-level features. The LowLevel variant only uses the MsCGA block to enhance low-level features, while Crack-MsCGA uses the MsCGA block only to enhance high-level features.

Among all variants, Crack-MsCGA achieved the highest mAP@50, with a computational cost only slightly higher than the WOA variant, which does not use the MsCGA block. This confirms that the theory also applies to crack detection. The LowLevel variant improved mAP@50 by only 0.1% compared to the WOA variant, while the AllLevel variant improved mAP@50 by just 0.3%. This is because the information learned by the attention mechanism can easily diminish the local features in large-scale features.

### 4.5. Comparative Experiments on Window Size of Local Window Attention Mechanism

In Section 3.2, we proposed a local window attention mechanism to compute short-range dependencies by aggregating the local features of pavement cracks within each window patch, with the window size set as the hyperparameter ws. In this section, we design comparative experiments to investigate the impact of different window sizes on asphalt pavement crack detection. Table 7 presents the detection accuracies on the DH807 dataset using different window sizes.

Firstly, all variants exhibit similar performance in detecting large-scale cracks. This is because each variant employs a CGA mechanism to capture intricate and variable global attention features by learning long-range contextual information across the entire image. These global attention features facilitate the detection of large-scale cracks.

Secondly, as the ws increases, the detection accuracy for medium-scale cracks gradually declines.

Finally, when ws is set to five, both small-scale and large-scale crack detection accuracies peak, and the average mAP@50 is significantly higher than that of the other variants. This indicates that a window size of 5 is optimal.

### 4.6. Ablation Experiment

The primary difference between Crack-MsCGA and other comparative models lies in the neck network architecture. It first employs MsCGA to enhance high-level features, and then fuses these features with low-level features. In this section, we design ablation experiments to examine the rationale behind the neck network structure in Crack-MsCGA.The experimental results are shown in Table 8. The “Fused Features” column indicates the features used in the neck fusion process, while the “MsCGA” column specifies whether the MsCGA attention mechanism is applied.

Firstly, we design a WOA variant by removing the MsCGA block from Crack-MsCGA to analyze its contribution. Compare to the WOA variant, Crack-MsCGA exhibits improvements in crack mAP@50 across all scales, achieving gains of 3.6%, 3.8%, and 4.2% for small-, medium-, and large-scale cracks, respectively. This suggests that MsCGA plays a pivotal role in achieving high-precision pavement crack detection in the Crack-MsCGA model.

Secondly, while most common pavement crack detection models integrate multi-level features (S2, S3, and S4), Crack-MsCGA only incorporates features from S3 and S4. To validate the rationality of this design, we developed an S3 variant that, compared to Crack-MsCGA, additionally fuses the lower-level S3 feature. Experimental results indicate that, compared to Crack-MsCGA, the S3 variant shows a decline in crack detection accuracy across all scales—most notably, a 3.4% drop for small-scale cracks. On one hand, this can be attributed to the backbone network’s use of a CNN for feature extraction; although the lower-level S3 features provide more detailed information, they also introduce substantial background noise during the fusion process. On the other hand, fusing S3 dilutes the crack-specific features that the MsCGA block learned.

From the perspective of computational complexity, a comparison between Crack-MsCGA and the S2 variant reveals that reducing the fusion of low-level features ($S_{2}$) enables Crack-MsCGA to reduce to 23.9 GFLOPs. Additionally, when compared to the S2 WOA variant, it is evident that the MsCGA block requires 1.4 GFLOPs of computational cost. Overall, our unique neck design reduces the network’s computational complexity by 22.5 GFLOPs while simultaneously improving the accuracy of pavement crack detection.

### 4.7. Conclusions

In this study, we focus on improving the detection accuracy of slight small-scale cracks with insignificant structures and variable morphologies. We propose a Multi-scale Cascaded Group Attention (MsCGA) mechanism to learn and fuse both short-range and long-range dependencies of pavement cracks at different scales. Based on MsCGA, we build a pavement crack detection model named Crack-MsCGA. Crack-MsCGA shows its promising potential in the task of pavement crack detection, achieving the highest accuracy with minimal computational cost and the fewest parameters at all scales and all categories.

In the future, we will study pavement crack detection based on 3D point cloud data collected by LiDAR, aiming to further improve the detection effect of small-scale road cracks.

## Figures and Tables

**Table 1:** Performance on China-M dataset.

Methods	AP@50(%) ↑				mF1
	Small	Medium	Large	Mean	
YOLOv5l	73.5	83.9	94.9	84.1	87.4
YOLOv5l-ST-BIFPN	73.7	83.0	93.8	83.5	88.8
UM-YOLOl	76.5	84.8	95.0	85.4	88.6
Faster-RCNN-R50	69.9	76.0	93.7	79.9	83.1
Deformable-DETR-R50	68.7	78.6	93.2	80.2	82.3
DDQ-DETR-R50	77.1	86.3	94.7	86.0	91.5
RT-DETR-R50	72.6	85.3	95.4	84.5	85.5
Crack-MsCGA (ours)	84.8	86.8	96.3	89.3	92.3

**Table 2:** Performance on DH807 dataset.

Methods	AP@50(%) ↑				mF1
	Small	Medium	Large	Mean	
YOLOv5l	35.4	59.7	70.2	55.1	66.7
YOLOv5l-ST-BIFPN	33.6	58.4	70.5	54.2	64.1
UM-YOLOl	31.8	60.3	66.7	53.0	63.4
Faster-RCNN-R50	25.2	55.1	65.2	48.5	57.3
Deformable-DETR-R50	26.4	38.3	61.1	42.0	47.6
DDQ-DETR-R50	30.6	61.6	65.1	52.4	62.9
RT-DETR-R50	36.7	67.8	69.0	57.8	67.4
Crack-MsCGA (ours)	42.7	65.4	72.7	60.3	69.9

**Table 3:** GFLOPs and parameters of Crack-MsCGA and other models.

Methods	GFLOPs ↓	Parameters (G) ↓
YOLOv5l	53.8	46.1
Faster-RCNN-R50	91.3	41.8
Deformable-DETR-R50	79.7	40.2
DDQ-DETR-R50	52.8	48.3
RT-DETR-R50	64.8	42.0
Crack-MsCGA (ours)	44.5	38.5

**Table 4:** FPS of Crack-MsCGA and the comparison models on different platforms under different floating point precisions, where FP32 represents the inference speed measured under a 32-bit floating point precision, while FP16 represents the inference speed measured under a 16-bit floating point precision.

Methods	NVIDIA RTX A6000		NVIDIA RTX 3060	
	FP32	FP16	FP32	FP16
YOLOv5l	182.0	294.2	68.6	122.3
Faster-RCNN-R50	96.3	146.5	38.9	60.5
Deformable-DETR-R50	86.7	124.1	32.6	56.8
DDQ-DETR-R50	115.3	196.4	46.3	84.6
RT-DETR-R50	105.4	189.0	40.7	78.2
Crack-MsCGA (ours)	136.6	242.9	55.4	107.6

**Table 5:** Performance with MsCGA and other attention mechanisms.

Methods	AP@50(%) ↑			
	Small	Medium	Large	Mean
WOA	81.2	83.0	92.1	85.4
CBAM [9]	77.6	83.0	94.0	84.9
DAttention [51]	80.9	84.9	94.0	86.6
AIFI [39]	81.7	83.7	94.5	86.6
FLA [52]	82.3	85.1	93.7	87.0
LSKA [53]	81.3	84.3	92.9	86.2
MsCGA (ours)	84.8	86.8	96.3	89.3

**Table 6:** Study on effectiveness of applying attention enhancement at different layers.

Methods	Attention Levels		GFLOPs ↓	mAP@50(%) ↑
	Low	High		
WOA	✗	✗	43.1	93.0
LowLevel	✓	✗	48.5	93.1
AllLevel	✓	✓	49.8	93.3
Crack-MsCGA	✗	✓	44.5	93.9

**Table 7:** Comparative experiments on window size of local window attention mechanism.

Window Size	AP@50(%) ↑			
	Small	Medium	Large	Mean
3	36.7	66.5	72.5	58.6
5	42.7	65.4	72.7	60.3
7	40.0	64.0	71.6	58.5
9	41.0	62.5	72.5	58.7

**Table 8:** Study on effectiveness of applying attention enhancement at different layers.

Methods	Fused Features			MsCGA	GFLOPs ↓	AP@50(%) ↑			
	$S_{2}$	$S_{3}$	$S_{4}$			Small	Medium	Large	Mean
WOA	✗	✓	✓	✗	43.1	81.2	83.0	92.1	85.4
S2	✓	✓	✓	✓	68.4	81.4	86.5	95.2	87.7
Crack-MsCGA (ours)	✗	✓	✓	✓	44.5	84.8	86.8	96.3	89.3

## Data Availability

The data that support the findings of this study are available from the corresponding author, B.H., upon reasonable request.
